# Evaluation of two-fold fully conditional specification multiple imputation for longitudinal electronic health record data

**DOI:** 10.1002/sim.6184

**Published:** 2014-04-30

**Authors:** Catherine A Welch, Irene Petersen, Jonathan W Bartlett, Ian R White, Louise Marston, Richard W Morris, Irwin Nazareth, Kate Walters, James Carpenter

**Affiliations:** a Department of Primary Care and Population Health, University College London (UCL)London, U.K.; b Department of Medical Statistics, London School of Hygiene and Tropical MedicineLondon, U.K.; c MRC Biostatistics UnitCambridge, U.K.; d MRC Clinical Trials Unit, Aviation HouseKingsway, London, U.K.

**Keywords:** multiple imputation, missing data, partially observed, longitudinal electronic health records

## Abstract

Most implementations of multiple imputation (MI) of missing data are designed for simple rectangular data structures ignoring temporal ordering of data. Therefore, when applying MI to longitudinal data with intermittent patterns of missing data, some alternative strategies must be considered. One approach is to divide data into time blocks and implement MI independently at each block. An alternative approach is to include all time blocks in the same MI model. With increasing numbers of time blocks, this approach is likely to break down because of co-linearity and over-fitting. The new two-fold fully conditional specification (FCS) MI algorithm addresses these issues, by only conditioning on measurements, which are local in time. We describe and report the results of a novel simulation study to critically evaluate the two-fold FCS algorithm and its suitability for imputation of longitudinal electronic health records. After generating a full data set, approximately 70% of selected continuous and categorical variables were made missing completely at random in each of ten time blocks. Subsequently, we applied a simple time-to-event model. We compared efficiency of estimated coefficients from a complete records analysis, MI of data in the baseline time block and the two-fold FCS algorithm. The results show that the two-fold FCS algorithm maximises the use of data available, with the gain relative to baseline MI depending on the strength of correlations within and between variables. Using this approach also increases plausibility of the missing at random assumption by using repeated measures over time of variables whose baseline values may be missing.

## 1. Introduction

Electronic health records of routinely collected clinical information are a valuable resource for epidemiological investigations and health care research. They offer many opportunities to answer questions in populations, which are otherwise difficult and expensive to study using standard observational studies and clinical trials. This includes research on individuals with severe mental illness, pregnant women, children and the very elderly people 1–5. The databases are a powerful data sources for research into cardiovascular diseases including coronary heart disease (CHD) and stroke 6,7. One example of electronic health records is the The Health Improvement Network (THIN) primary care database. It consists of clinical records that capture information on medical diagnoses, symptoms and prescribed medication as well as information on health indicators through routine consultations with a general practitioner or health care professionals. THIN includes electronic health records for more than 11 million patients registered with over 500 General Practices across the UK, some of which date back to the early 1990s. Most patients have records of several consultations, from the point of registering with the practice to the time they leave, providing a longitudinal record of health data (mean follow-up time is 6.7 years) 8. In the first year after registration with the practice, patients typically have a record of past and current medical history and some have health indicators such as height, weight, blood pressure, alcohol and smoking status measured 9. Thereafter, this information is only recorded intermittently and if directly relevant to the clinical management of the patient. For example, patients with previous cardiovascular events, or those who are at risk of these, are much more likely to have several, regular measurements of health indicators recorded compared with patients without such events. Indeed, since 2004, patients diagnosed with any one of a number of chronic conditions or diseases are monitored at regular intervals as a part of the NHS care delivery plan for UK general practice (Quality Outcome Framework) 10. Despite this, previous research shows nearly 40% of patients are missing blood pressure or weight measurements in the first year after registration 9. Such patterns of clinical observations are typical in databases of this kind and may be an obstacle to their use in epidemiological research, which requires accurate records of key health indicators over time.

Within medical research, multiple imputation (MI) 11 is increasingly regarded as the standard method to analyse missing data 12. MI generates complete data sets. In each, missing values are appropriately imputed from the estimated distribution of the missing data given the observed data. Each of these completed data sets is analysed in the standard way, that is, using the method that would have been used if there were no missing data. The results are combined for final inference using Rubin′s rules (see, for example, 13, Chapter 2).

Most implementations of MI are designed with a simple rectangular structure and do not take temporal ordering of the data into account. Electronic health records often contain longitudinal and dynamic records, with intermittent patterns of missing data and many variables recorded each time. This makes the missing data problem more complex than other study settings such as clinical trials and standard cohort studies. One approach is to divide the data into blocks of time and implement MI independently at each time block. Consequently, correlations between measurements at different time blocks are distorted in the imputed data, and longitudinal information in the observed data is ignored. In general, this approach yields biassed parameter estimates, particularly if interest lays in the longitudinal evolution of health indicators. An alternative approach is to include all of the time blocks in the same MI model, with distinct variables for measurements of each health indicator at each time block. This approach may work well for a few time blocks, but it does not exploit the temporal ordering of the measurements. As variables and time blocks increase, this approach may break down because of problems of co-linearity in predictors and over-fitting. This is likely to be a particular problem for categorical variables. Conversely, methods that take full account of the hierarchical and longitudinal structure (13, Chapter 9) are currently computationally intractable in these settings.

A number of studies have investigated MI in longitudinal records but in specific or simpler settings. Liu and Zhan 14 considered data with a monotone missingness pattern; Vinogradova *et al*. and Sarraceno *et al*. 15,16 imputed missing data at a single time point; Grittner *et al*. 17 and Lewis *et al*. 18 used small data sets with few time points and thus were able to include measurements at all time points in the imputation model without any computational issues arising. Howard *et al*. 19 and Wood *et al*. 20 focused on imputing only the outcome.

Nevalainen *et al*. 21 proposed a new implementation of MI, termed two-fold fully conditional specification (FCS), which exploits the temporal relationship between variables in longitudinal data with a potentially non-monotone missingness pattern. The two-fold FCS algorithm only conditions on measurements local in time to address the problems raised. Specifically, it visits each time block in turn and imputes data at this time block, say *t*, taking into account variables measured at times *t*, and adjacent time blocks (*t* − 1) and (*t* + 1). The two-fold FCS algorithm iterates through all the time blocks many times and thus carries information through the longitudinal records.

Nevalainen *et al*. illustrated the potential of this approach using data simulated from a case-control study with measurements collected at just three time points with up to 40% of records missing. However, it is unclear how the two-fold FCS algorithm performs in the more complex setting of longitudinal electronic health records with a substantially higher proportion of missing data and more than three time points. Thus, in this paper, we describe and report the results of a novel simulation study to further evaluate the two-fold FCS algorithm with the aim of investigating the suitability of the two-fold FCS algorithm for imputation of longitudinal electronic health records. We generated the data for the simulation study on the basis of the dynamic patterns of data recording in UK primary care derived from THIN. We focus on MI of a few variables that constitute a mixture of continuous and categorical health indicators typically included in epidemiological studies as risk predictors or confounders. After generating a full data set, we made approximately 70% of the data in selected variables missing completely at random (MCAR) in each of ten time blocks. We applied a substantive time-to-event model, which mimics essential features of a cohort study, to examine risk for CHD. We compare efficiency and coverage of confidence intervals for estimated coefficients from (i) a complete records analysis, (ii) MI of data in the baseline time block alone and (iii) MI of the longitudinal data using the two-fold FCS algorithm.

The plan for the article is as follows: Section 2 describes the two-fold FCS algorithm in detail, whereas Section 3 gives details of the data generation and simulation process. The results are presented in Section 4, and we conclude with a discussion in Section 5.

## 2. The two-fold fully conditional specification algorithm

### 2.1. Setup

We assume longitudinal records exist for 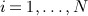 independent individuals with a time-to-event outcome, **Y**_*i*_ = (*Δ*_*i*_,*T*_*i*_)^*T*^, where Δ_*i*_ indicates whether the individual experienced the event of interest (Δ_*i*_ = 1) or was censored (Δ_*i*_ = 0) and *T*_*i*_ is the earlier of the time-to-event and time-to-censoring. Let 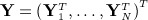 denote the 2*N* × 1 vector of time-to-event data. We assume 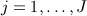 covariates exist with values at *t* = 1, … ,*T* (typically equally spaced) time blocks. Let *X*_*i*,*j*,*t*_ denote the value of variable *j* for individual *i* at time *t*. Let 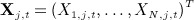 be the *N* × 1 vector of values of covariate *j* at time *t* and 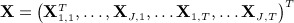 denote the *NJT* × 1 vector of covariates. Lastly, let **X**_*mis*_, **X**_*obs*_ denote the subvectors of **X** corresponding to the missing and observed values, respectively. We assume that data are missing at random (MAR).

### 2.2 Joint model multiple imputation

At its heart, MI has a joint model for the data, which consists of **Y**, **X** and a vector of response variables indicating which components of **X** are observed and which are missing. Assuming MAR and parameter distinctiveness, there is no need to model this response indicator, and we can express the joint model as 

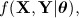
1
where ***θ*** is a parameter vector. In its original form, MI proceeds by drawing *K* independent realisations from the posterior distribution *f*(**X**_*mis*_,***θ*** | **X**_*obs*_,**Y**), typically using MCMC, and then discarding the draws of ***θ*** to give *K* imputed data sets 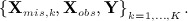 The substantive model is fitted to each imputed data set in turn, using the usual full data procedure, and the results are summarised for final inference using Rubin′s rules (for details see 13, Chapter 2).

### 2.3 Full conditional specification multiple imputation

The FCS approach to MI 22 approximates the joint model MI procedure by, in turn for each variable 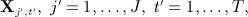 which constitutes an *iteration*

1. regressing 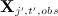 on all other variables 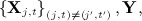 where missing values in the covariates (of the imputation model) take their current imputed values. Note this is fitted to the subset of individuals for whom 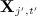 was originally observed;
2. using the regression model to generate one imputation of each missing value in 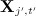.

Fully conditional specification is initiated by imputing missing values in each variable **X**_*j*,*t*_ by sampling with replacement from observed values of **X**_*j*,*t*_.

Starting from the initial values, FCS completes a few iterations, typically 20 23, and the current imputed and observed data become the first imputed data set. FCS completes a few further iterations before taking the current imputed and observed values as the second imputed data set. The number of imputation updates is chosen so that the imputed data sets are independent.

Similarities between MI using FCS and MI from a joint model are only established in certain circumstances for multivariate normal and multivariate categorical data (13, Chapters 3, 4). However, many simulation studies (e.g. 23) indicate FCS performs well in practice. One reason FCS is popular is because it is flexible, and different regression model types are used to impute different variable types. FCS thus is a natural candidate to impute the longitudinal data described previously.

### 2.4 Imputing longitudinal data

As outlined in Section 1, several drawbacks exist to apply FCS so each health indicator/time block combination is included as a separate covariate. This is evident when we consider the many covariates ((*JT* − 1) + 2) included in each FCS regression model. With a few time blocks, it is a feasible approach, but with many time blocks and variables, as mentioned previously, one may encounter co-linearity and over-fitting issues, particularly for categorical variables 24.

An alternative option is to impute separately for each time block, that is, apply the standard FCS separately for each 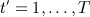, following which imputed data sets for each *t* ′  are attached. As mentioned previously, this approach generally leads to biassed estimates because the longitudinal correlation structure is distorted. Moreover, typically values of health indicators are correlated over time so that such an approach ignores the useful information in observed measurements available at other times. This makes the imputation method less efficient than other imputation methods, which do use the information observed at other times.

### 2.5 The two-fold fully conditional specification algorithm

As a compromise between the two approaches, Nevalainen *et al*. 21 proposed the two-fold FCS algorithm. The key idea is the imputation model used to impute missing values at time *t* ′  only condition on values at time *t* ′  and the preceding and following time blocks. This creates the following algorithm:

The two-fold FCS algorithm consists of the following steps. First, a time window width *τ* (typically 1,2 or 3) is chosen. Then, for 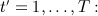


*Within-time:*

Apply standard FCS imputation, with *b*_*W*_ iterations, to the variables 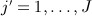 at time *t* ′ , using regressions

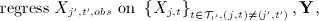
2imputing missing data in 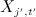 at each step, where 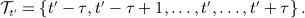 This means that, at time *t* ′ , each of the *J* variables is imputed conditional on values of variables in the window width 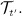 Thus, observed and previously imputed values before and after time *t* ′ , which fall within the specified window width, are predictors in the imputation model and not imputed at this stage.

*Among-time:*

The within-time iteration is applied to each time block 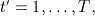 typically (but not necessarily) in time order. One among-time iteration is complete when all time blocks are visited. The algorithm is repeated for *b*_*A*_ among-time iterations.

Data are unavailable at all time blocks within the window width close to the beginning and end of the follow-up, which extends below *t* ′  = 1 or above *t* ′  = *T*. In this case, we restrict to time blocks with available observations within the window width.

In their proposal, Nevalainen *et al*. 21 only impute time-dependent variables. In our implementation of the algorithm, we included an additional step to impute missing values for time-independent variable at the start of each among-time iteration, conditional on outcome *Y* and other fully observed time-independent variables. Additionally, we chose to condition on time-dependent variables (either with missing data or fully observed) at a particular ‘baseline’ time block, which can be different for each individual. Fully observed time-dependent variables are also included as predictors in imputation models of time-dependent variables, but only values within the window width are included. Similarly, we also condition on fully observed time-independent variables, for example, sex.

## 3. Data generation and simulation process

To evaluate the performance of the two-fold FCS algorithm, we designed a simulation study modelled closely on THIN data. The details of the data generating process are described in the succeeding text. For each of 1000 independent simulations, we

1. generated a complete set of observations for 5000 patients;
2. fitted our substantive model to this full data, recording parameter estimates and standard errors;
3. made some of the health indicator measurements MCAR;
4. imputed the missing data with the relevant algorithm under evaluation;
5. fitted our substantive model to each imputed data set, before combining the results using Rubin′s rules 11 and recording the resulting imputation-based parameter estimates and standard errors.

### Data generation mechanisms

Each simulated data set consisted of a cohort of 5000 patients (slightly smaller than the average patient list size in a UK primary care practice). Data were generated for ten time blocks, corresponding to 10 years (e.g. 2000–2009). For each patient in each data set, we simulated the following categorical and continuous health indicator and patient characteristic variables: newly registered (in 1999) in the general practice or registered earlier, smoking status, age at baseline, deprivation (quintiles of Townsend score index), systolic blood pressure, weight and anti-hypertensive drug treatment. These variables were chosen to illustrate key risk factors for CHD.

We used two different data generating mechanisms for smoking status. First, we used an approach (data generation mechanism I) whereby smoking status was generated at the first time block and kept constant over time. Second, we used an approach (data generation mechanism II) whereby approximately half of the smoking status records were non-smoker in the first time block and kept constant over time. For the remaining patients, smoking status records alternate between current smoker and ex-smoker over the ten time blocks. The second data generating mechanisms purpose was to evaluate the feasibility of imputing time-dependent longitudinal categorical data.

The outcome of interest was generated from an exponential time-to-event model, with hazard depending on the health indicator values at the first time point. Further details of the data generation mechanisms are in the Appendix.

### Parameters values used for the data generation mechanisms

Each of the data generation steps (Appendix) required values of associated model parameters. We derived these values from a cohort of patients from THIN considered the ‘true’ values in the simulation study (although we do not intend any clinical inference from the subsequent analyses). The data sample from THIN used in this analysis consisted of male patients aged between 40 and 89 years in the first time block (2000) and with no records of CHD events before the start of the second time block (1 January 2001). Time-to-event was calculated as the time from 1 January 2001 to the date of the first CHD diagnosis.

In this sample data from THIN,some weight, systolic blood pressure and smoking status measurements were missing. We therefore estimated the parameters of the models used to generate the simulated data sets using those with complete records (i.e. patients with no missing data across all variables under consideration).

### Missingness mechanism

We imposed MCAR missingness for each simulated data set. Age, social deprivation and anti-hypertensive drug treatment were fully observed. For the remaining variables, weight, systolic blood pressure and smoking status, data were missing in any year unless (i) the patient ‘consulted’ and (ii) given they consulted, the variable was ‘recorded’.

For each patient and year, we simulated a binary indicator ‘consulted’, with probability 0.3 of consultation. Conditional on ‘consulted’ in a given year, systolic blood pressure, weight and smoking status (for data generating mechanism II) were made (independently) missing with probability 0.05. For most individuals, data are therefore either all missing or all observed for the time-dependent variables in a given time block (i.e. consultation). However, for a small proportion of individuals, some (but not all) of the variables are observed in a time block (i.e. consultation). Under this mechanism, for example, the expected years between weight observations is 1 / (0.3 × 0.95) = 3.5 years. This seems high but is broadly consistent with missing data in electronic health records such as THIN.

### Estimation methods

We estimated the exponential time-to-event model parameters, which is the substantive model of interest in many epidemiological studies. For each simulated data set, we first estimated the parameters of the exponential time-to-event model using the full data, by maximum likelihood. Next, we fitted the model using the complete records. This consisted of patients with no missing risk factor values at time *t* = 1 because the covariates in the time-to-event model were measurements of health indicators at *t* = 1.

We multiply the imputed missing data using three approaches. On the basis of the results of White and Royston 25, the event indicator and an estimate of the baseline cumulative hazard function should be included as covariates when imputing covariates in survival models. Because we generated times from an exponential model, the cumulative hazard is proportional to time, and thus, we included the event indicator and time-to-event or censoring as predictors in all imputation models. We implemented the following three imputation approaches:

1. Applied standard FCS imputation to data from all time blocks (2000–2009), conditioned on the event indicator and time-to-event or censoring. Each imputation model conditioned on health indicators measurements at all other time blocks, plus measurements of health indicators other than the one being imputed at the current time block. We refer to this as ‘FCS’.
2. Applied standard FCS imputation to the data recorded in the first time block, (the baseline year 2000), ignoring data from all other years. Thus, missing data in this time block were imputed conditional only on other measurements recorded in this block and the CHD event indicator and time-to-event or censoring. We refer to this as ‘baseline FCS imputation’.
3. Applied the two-fold FCS algorithm to data from all time blocks (2000–2009), again conditioned on the event indicator and time-to-event or censoring. For this study, we used *b*_*w*_ = 5 within-time iterations, *b*_*a*_ = 20 among-time iterations and a *τ* = 1 time window.

For each imputation approach, we generated five imputed data sets and combined the results using Rubin′s rules. We applied all three imputation approaches to the data generated from data generating mechanism I, with time-independent smoking status.

We applied the two-fold FCS algorithm to data generated from data generating mechanism II (non-smokers remained non-smokers at all times, but smoking status for the remaining patients could vary between current smokers and ex-smokers). We used a semi-deterministic approach to simplify the imputation to a ‘binary’ problem. Hence, before we applied the two-fold FCS algorithm, we identified patients only ever observed as a non-smoker (only had the non-smoker smoking status recorded, never ex-smoker or current smoker) and deterministically imputed them as a non-smoker at all time blocks. The resulting data set was imputed using the two-fold FCS algorithm, whereby missing values in the smoking status variable were imputed only as current smokers or ex-smokers. We adopted this approach to overcome the problem of a patient who is non-smoker at a later time block being imputed as a current smoker at an earlier time block (Section 5).

To express gains and losses in relative efficiency (information), we calculated the ratio of the empirical variance of the full data estimator to the empirical variance of each of the other (partial data) estimators, also expressed as reductions in effective sample size, relative to analysis of the full data.

We performed analyses using Stata version 11.2 and 12.2 (StatCorp LP, Texas, USA) (www.stata.com) , and we wrote a separate Stata command ‘twofold’ to perform the two-fold FCS algorithm 26. This is available from add http://www.ucl.ac.uk/pcph/research-groups-themes/thin-pub/missing_data. The ‘twofold’ command uses MI IMPUTE CHAINED, which is a part of the official Stata command library.

## 4. Results

The full FCS approach, conditioned on measurements of health indicators at all time blocks, failed to run successfully on approximately 25% of data sets and so is not considered further.

Table 1 shows the log hazard ratios from fitting the model of interest to THIN cohort and the average estimates (1000 replications) from the full data, complete records, baseline FCS imputation and two-fold FCS algorithm, for data generating mechanism I and II. The parameter estimates from complete records, baseline FCS imputation and the two-fold FCS algorithm all had practically negligible bias (results not shown). We expected the complete records analysis to be unbiassed because the data were MCAR. The results from baseline FCS imputation and imputation using two-fold FCS algorithm were also unbiassed, which suggests that the conditional imputation models were (at least approximately) correctly specified.

**Table 1:** Log hazard ratios from fitting an exponential model to predict risk of coronary heart disease: estimates from a data sample from The Health Improvement Network, followed by the average of estimates across 1000 simulations based on full data, complete records,‘baseline fully conditional specification (FCS) multiple imputation’, and missing values imputed using the two-fold FCS algorithm.

Variables		THIN cohort	Full data	Complete records	Baseline FCS imputation	Two-fold FCS	
						DGM I	DGM II	DGM I	DGM II
Townsend	1	Reference					
deprivation	2	0.1520	0.1503	0.1425	0.1497	0.1498	0.1588
score quintile	3	0.2377	0.2367	0.2431	0.2366	0.2366	0.2422
	4	0.2433	0.2400	0.2279	0.2391	0.2401	0.2535
	5	0.4034	0.4024	0.3935	0.4020	0.4023	0.4017
Weight (kg)		0.0019	0.0019	0.0015	0.0016	0.0019	0.0017
Systolic blood pressure (mmHg)		0.0048	0.0049	0.0051	0.0048	0.0051	0.0053
Anti-hypertensive drug treatment		0.2935	0.2868	0.2852	0.2897	0.2855	0.2915
Smoking status	Non-smoker	Reference					
	Ex-smoker	0.0679	0.0692	0.0633	0.0672	0.0579	0.0567
	Current smoker	0.2386	0.2385	0.2307	0.2342	0.2325	0.2261
Age group (years)	40–44	− 1.2820	− 1.2872	− 1.3167	− 1.2869	− 1.2880	− 1.2890
	45–49	− 1.0632	− 1.0652	− 1.0892	− 1.0655	− 1.0662	− 1.0623
	50–54	− 0.6402	− 0.6392	− 0.6467	− 0.6398	− 0.6408	− 0.6330
	55–59	− 0.3589	− 0.3597	− 0.3700	− 0.3598	− 0.3605	− 0.3536
	60–64	− 0.2485	− 0.2473	− 0.2545	− 0.2480	− 0.2481	− 0.2423
	65–69	− 0.0396	− 0.0416	− 0.0470	− 0.0418	− 0.0409	− 0.0348
	70–74	Reference					
	75–79	0.1108	0.1039	0.1116	0.1043	0.1057	0.1129
	80 +	0.1387	0.1421	0.1255	0.1383	0.1414	0.1368
Constant term		− 5.1993	− 5.2297	− 5.2550	− 5.2098	− 5.2552	− 5.2833

DGM, data generation mechanism; FCS, fully conditional specification; THIN, The Health Improvement Network.

Table 2 shows the analytical and empirical standard errors from each method and shows good agreement for all methods. Confidence interval coverage for all methods was approximately equal to its nominal 95% level (not shown). As is typically seen in the absence of the inclusion of auxiliary variables, baseline FCS imputation resulted in substantially smaller standard errors than complete records analysis for the coefficients of the fully observed covariates but not for covariates with missing data. Two-fold FCS algorithm gave improved efficiency compared with baseline MI for the coefficients of the time-dependent covariates with missing data, with the largest gain for weight. This gain can be attributed to the two-fold FCS algorithm using information in longitudinal measurements of the time-dependent variables. The relatively large gain in efficiency for the weight coefficient is because this variable is most strongly correlated over time (the correlation between weight in 2000 and weight in 2001 was 0.96, whereas the correlation for systolic blood pressure in 2000 and systolic blood pressure in 2001 was only 0.65). In contrast, two-fold FCS algorithm did not gain efficiency (relative to baseline FCS imputation) for the coefficient of the time-independent smoking status variable with missing data (data generating mechanism I). However, when smoking status was time-dependent (data generating mechanism II), the two-fold FCS algorithm gained substantial efficiency over both analysis of complete records and baseline FCS imputation with a reduction in standard errors of around 35%

**Table 2:** Standard errors (SE) from fitting the exponential model to predict risk of coronary heart disease to the full simulated data and SE and empirical SE found from fitting the exponential model to the complete records analysis after full simulated data sets were changed to missing, imputed data using baseline fully conditional specification (FCS) imputation and imputed data using the two-fold FCS algorithm.

Variables		Full data	Complete records		Baseline FCS imputation		Two-fold FCS-DGM 1		Two-fold FCS-DGM 2	
		SE	SE	Empirical SE	SE	Empirical SE	SE	Empirical SE	SE	Empirical SE
Townsend	1	Reference								
deprivation	2	0.1286	0.2528	0.2472	0.1290	0.1304	0.1281	0.1295	0.1249	0.1267
score quintile	3	0.1326	0.2621	0.2630	0.1349	0.1366	0.1340	0.1363	0.1266	0.1322
	4	0.1419	0.2793	0.2817	0.1442	0.1462	0.1432	0.1473	0.1341	0.1404
	5	0.1541	0.3087	0.3133	0.1600	0.1627	0.1586	0.1606	0.1535	0.1563
Weight (kg)		0.0032	0.0064	0.0062	0.0063	0.0067	0.0041	0.0041	0.0043	0.0041
Systolic blood pressure (mmHg)		0.0026	0.0055	0.0055	0.0054	0.0056	0.0050	0.0049	0.0053	0.0050
Anti-hypertensive drug treatment		0.0957	0.1923	0.1933	0.1113	0.1133	0.1060	0.1033	0.1109	0.1060
Smoking status	Non-smoker	Reference								
	Ex-smoker	0.1074	0.2117	0.2153	0.2104	0.2289	0.2064	0.2180	0.1330	0.1326
	Current smoker	0.1143	0.2260	0.2302	0.2221	0.2410	0.2161	0.2312	0.1538	0.1489
Age group (years)	40–44	0.2311	0.4659	0.4936	0.2484	0.2538	0.2425	0.2448	0.2410	0.2409
	45–49	0.2137	0.4321	0.4395	0.2287	0.2328	0.2231	0.2236	0.2228	0.2220
	50–54	0.1872	0.3673	0.3762	0.1954	0.2021	0.1907	0.1962	0.1871	0.1895
	55–59	0.1734	0.3576	0.3657	0.1867	0.1817	0.1832	0.1791	0.1827	0.1825
	60–64	0.1783	0.3589	0.3706	0.1846	0.1848	0.1819	0.1831	0.1753	0.1815
	65–69	0.1764	0.3545	0.3671	0.1802	0.1815	0.1790	0.1801	0.1727	0.1784
	70–74	Reference								
	75–79	0.1914	0.3885	0.3879	0.1998	0.1959	0.1966	0.1955	0.1936	0.1957
	80 +	0.2028	0.4122	0.4272	0.2132	0.2106	0.2076	0.2071	0.2139	0.2103
Constant term		0.4554	0.9369	0.9516	0.8885	0.9092	0.7481	0.7406	0.7930	0.7371

DGM, data generation mechanism; FCS, fully conditional specification.

For the weight coefficient, the loss of information from the full data analysis to complete records, baseline FCS imputation and imputation using the two-fold FCS algorithm is, respectively, 73%, 77% and 39%. Thus, for the weight coefficient, complete records analysis was similar to baseline FCS imputation, with both loosing about 3/4 of the information relative to the full data. In other words, a sample of 1000 individuals is reduced to an effective sample size of around 230 for baseline FCS imputation and 270 for complete records analysis, whereas it is reduced to 600 if we impute using the two-fold FCS algorithm.

Our Stata implementation of the two-fold algorithm took (on average) 4 min to create each imputed data set using 20 among-time and five within-time iterations. The baseline FCS approach took 6 s per imputation using the default 10 iterations. The difference in speed can be attributed to the fact that the two-fold algorithm fits many more imputation models than the ‘baseline FCS’ approach and that the total number of iterations used per imputation was larger for two-fold.

## 5. Discussion

In this study, we evaluated the performance of the two-fold FCS algorithm first proposed by Nevalainen *et al*. 21. We simulated 1000 data sets of 5000 patients using parameter estimates from a THIN male cohort. We chose variables representing ‘typical’ health indicators in many epidemiological and health research settings. Some were continuous (blood pressure and weight), while others were categorical (smoking status). We made approximately 70% of the values in weight, blood pressure and smoking status MCAR at each time block and compared the bias and efficiency of the two-fold FCS algorithm with complete records analysis and baseline FCS imputation in a simple time-to-event model.

A FCS imputation approach failed for approximately 25% of the simulated data sets, because of co-linearity issues. In contrast, missing data imputed using the two-fold FCS algorithm gave essentially unbiassed estimates and made use of the longitudinal information, resulting in material gains in efficiency for the coefficients of time-dependent variables with missing data, relative to the baseline FCS imputation. The efficiency gain was greatest for the weight variable, likely because of its high within-subject correlation over time. In contrast, the systolic blood pressure coefficient was less highly correlated over time so the gain in efficiency was smaller. When smoking status was time-dependent, we again found large efficiency gains through using the two-fold FCS algorithm compared with baseline FCS imputation.

There was good agreement between empirical and imputation standard errors (calculated using Rubin′s rules) and provides further evidence in support of the arguments presented in 13, Chapter 2, in favour of the reliability of Rubin′s rules.

To impute longitudinal categorical data such as smoking status is particularly challenging. First, using a multinomial imputation model at each time block may become unstable if a large amount of data is missing. Second, some time-dependent categorical variables have restrictions on transitions over time. Smoking status can not transition from ex-smoker or current smoker statuses to non-smoker status, and it is not readily apparent how best to accommodate this restriction within the two-fold FCS algorithm. In our second data generating mechanism, smoking status is time-dependent but assumed that those who were initially non-smokers remained so throughout follow-up. This may be a reasonable assumption if individuals are above a certain age, because most smokers start before the age of 30 years. However, depending on the age range and duration of the study, non-smokers may become current smokers and then subsequently ex-smokers, which contradicts our assumption. In some situations, it may be possible for the longitudinal MI model to account for these conditional changes over time. Further, we made another simple assumption that all non-smokers would all have one or more non-smoker records. However, we recognise that a minority of non-smokers were never observed as non-smokers, who we (incorrectly) imputed as current smokers or ex-smokers throughout their follow-up.

We evaluated the two-fold FCS algorithm using a relatively simple substantive baseline model using a cohort approach examining the associations between variables in the first time block (year 2000) and subsequent CHD events. This mimics the common design in epidemiological research and derivation of predictive models. Further investigation is warranted to examine the two-fold FCS algorithm for alternative substantive models, in particular those in which the longitudinal evolution of variables is of interest and/or time-to-event models with time-dependent covariates.

We chose a scenario where approximately 70% of the records were missing in each time block. This may seem a high proportion, and some investigators may feel uncomfortable imputing this proportion of a variable. However, this is a similar situation to the analysis of electronic health records 9. It is thus reassuring that the two-fold FCS algorithm successfully runs even with such a large proportion of missing data. However, it is important to recognise that with very high proportions of missingness, the validity of inferences will be much more sensitive to misspecification of imputation models, and so we urge researchers to be suitably cautious when attempting to impute such large fractions of variables.

In our simulations, our data generation mechanism created values, which were MCAR. In practice, the MCAR assumption will often not hold. However, our focus in this paper was on the performance of the two-fold algorithm in comparison with both complete case analysis and ‘baseline’ imputation. The precise form of the missingness mechanism should not affect this comparison, provided it is ignorable. Further, the advantage of MCAR in our context is that the complete records analysis is unbiassed, so we can make direct comparisons with the efficiency of the complete records and various MI approaches. An important consideration is that because the two-fold FCS algorithm uses repeated measures over time of variables whose baseline values may be missing, the plausibility of the MAR assumption is arguably greater when longitudinal information is conditioned on. In particular, this is the case if the ‘baseline’ time for the substantive model is chosen at a time several years after a patient registered with the general practice.

As described in the Abstract, previous applications of MI to longitudinal data have usually considered somewhat simpler settings. Nevalainen *et al*. 21 who initially proposed the two-fold FCS algorithm only had three time points and up to 40% missing data. Therefore, this study is the first to demonstrate potential ways forward in MI of longitudinal records with a large number of time blocks and large proportions of missing data. Not only does the two-fold FCS algorithm offer a potential solution to the key issue of over-parameterisation of imputation models, which arises with this type of complex longitudinal data (see also 13, Ch 8), it also recovers longitudinal information and thus increases the precision of the estimates.

The major advantage of the two-fold FCS algorithm is it conditions on measurements local in time, and measurements outside those local measurements are feeding into the local imputation indirectly via the repeated among time imputations, which simplifies the imputation models. If measurements outside the chosen time window are conditionally associated with the measurements being imputed, estimates from imputations generated by two-fold FCS algorithm are expected to be biassed, in general. The data generating mechanism used in our simulation study violated the conditional independence assumption because it conditioned on measurements at all earlier time points when simulating values for weight and systolic blood pressure. Despite this, estimates based on imputations from the two-fold algorithm were essentially unbiassed for the survival substantive model used.

The plausibility of the conditional independence assumption made by the two-fold approach will clearly vary considerably from study to study, with factors affecting its plausibility including the frequency of longitudinal measurements, what quantities are being measured longitudinally and the disease under study. A simple approach to assess the plausibility of the assumption made by the two-fold approach is to fit, using complete case analyses, regression models, which include measurements outside of the selected time window. If these indicate that these measurements are independently associated with values at a given time, this suggests that the assumption does not hold. In some cases, this might be resolved by increasing the width of the time window, while in others, measurements at distant times may have independent associations, suggesting the two-fold approach is not appropriate. Further research is thus warranted to examine how realistic the conditional assumption is in practice.

When the number of within-time iterations is set to one, the two-fold algorithm is equivalent to a standard FCS approach with a particular set of imputation equations. Whether the doubly-iterative algorithm is beneficial (relative to using one within-time iteration), in respect of speed of convergence to the stationary distribution, warrants further investigation.

Finally, throughout, we assumed the outcome *Y* is readily conditioned on in the imputation models for time-dependent variables. For substantive models, which contain non-linear effects or interactions between risk factors, it may be difficult or impossible to impute compatibly 27. There is thus interest in exploring whether methods recently developed for imputing covariates compatibly with a given substantive model can be incorporated into the two-fold FCS algorithm.

## 6. Conclusion

Multiple imputation is increasingly considered a standard method to analyse missing data in medical research. However, most implementations of MI cannot account for the temporal structure in large longitudinal data. In this study, the two-fold FCS algorithm was successfully applied to simulated data that mimic data observed in longitudinal health records. The method is potentially applicable to other types of longitudinal records with intermittent missing data. The results show that the two-fold FCS algorithm maximises the use of data available, with the gain relative to baseline MI depending on the strength of correlations within and between variables. Using this approach also increases plausibility of the MAR assumption by using repeated measures over time of variables whose baseline values may be missing.

## Appendix A: Data generation mechanisms

### Data generation mechanism I

We now describe the first data generation mechanism. For each patient , we

1. marked patients *i* as registering at the general practice in 1999 with probability *p*_1_ and denoted this by setting a binary variable *reg*_*i*_ = 1; otherwise, they registered before 1999, and *reg*_*i*_ = 0;
2. generated baseline age (i.e. age in 2000) and denoted *age*_*i*_ with values *a* = 1, … ,10, corresponding to 5-year age categories from 40 to 89 years, using probabilities *q*_0,*a*_ for patients registered before 1999 (*reg*_*i*_ = 0) and probabilities *q*_1,*a*_ otherwise;
3. generated smoking status (time-independent) and denoted *smoke*_*i*_, from a multinomial logistic model conditional on age category in 2000:
  where *b* = 1 (non-smoker - reference category),2 (ex-smoker)* *and* *3 (current smoker);
4. generated social deprivation (Townsend score quintile, which takes values from 1 (least deprived) to 5 (most deprived)) and denoted *townsend*_*i*_, from an ordinal logistic regression model conditional on registration (1999 or earlier), age category in 2000 and smoking status:
  where *c* = 1, … ,5, with *c* = 1 as the reference category;
5. generated time-dependent systolic blood pressure and weight measurements and denoted *systolic*_*i*_ and *weight*_*i*_, respectively, for calendar years 2000 to 2009 (denoted ). For time block *t*, we generated these by
  where  and .
6. we generated binary time-dependent anti-hypertensive drug treatment variables and denoted *drug*_*i*,*t*_ for time *t*, from logistic regression models:
7. we generated a time to CHD event outcome *T*_*i*_ from an exponential distribution (constant hazard) with log hazard equal to
  Patients with a simulated value *T*_*i*_ greater than 10 were censored at time 10. Approximately, 10% of the patients in the simulated data had a CHD event within the 10 time blocks. For the purpose of this simulation study, transfer out of the practice and death from other causes were ignored.

### Data generation mechanism II

We also used a second data generation mechanism, identical to the first except for the way smoking status that was generated and conditioned on. Specifically, smoking status in the first time block (year 2000), *smoke*_*i*,1_, was generated using step 3 as described previously. Patients who were non-smokers in that time block were marked as non-smokers for each subsequent year of follow-up. For the remaining patients (smokers or ex-smokers) for , we generated

The remaining steps continued as for data generating mechanism I, except that when generating the values of the other time-dependent variables at time *t*, smoking status at time *t*,* smoke*_*i,t*_ was conditioned on. Townsend score and the time to CHD were generated conditional on smoking status at time *t* = 1.
